# Arginine at the host-pathogen interface

**DOI:** 10.1128/iai.00612-24

**Published:** 2025-07-03

**Authors:** Brooke E. Ryan, Laura A. Mike

**Affiliations:** 1 Medical Microbiology and Immunology, University of Toledo 7923, Toledo, Ohio, USA; 2 Department of Medicine, Division of Infectious Diseases, University of Pittsburgh 199716, Pittsburgh, Pennsylvania, USA; University of California at Santa Cruz, Santa Cruz, California, USA

**Keywords:** arginine, gene regulation, biofilm, toxins, secretion systems, ArgR, AhrC, swarming, metabolic regulation

## Abstract

Nutrient availability shapes the course of infection. Arginine, a conditionally essential amino acid, plays a crucial role in both host immune defense and pathogen metabolism. As a precursor for nitric oxide production, arginine supports immune functions in multiple immune cell types to control infections. However, it also serves as a signal for pathogens that promotes bacterial survival and growth. A plethora of recent studies have shown that arginine functions not only as a metabolic substrate but also as a key environmental cue that can alter cyclic diguanylate levels. Arginine availability regulates multiple bacterial processes in both Gram-positive and Gram-negative species including toxin production, biofilm formation, Type III secretion system, swarming, persistence, and immune evasion. In this way, arginine levels can shape how pathogens behave within the host environment. This review examines how fluctuations in arginine levels across different host niches influence microbial pathogenesis and highlights the complex interplay between arginine availability and bacterial behavior. Understanding the role of arginine in host-pathogen interactions may provide new therapeutic strategies to combat infections by targeting bacterial responses to this crucial nutrient.

## INTRODUCTION

Nutrient availability plays a critical role in regulating microbial virulence and ultimately influencing the course of infections. Among various nutrients, arginine, a conditionally essential amino acid in humans, stands out for its dual role in host defense and pathogen metabolism (1, 2). Arginine is essential for host immune functions, particularly in the production of nitric oxide (NO) by NO synthases (NOS), which plays a key role in immune defense by acting as an antimicrobial agent (3, 4). The role of arginine in host immune function has been reviewed extensively in Gogoi et al. (2), so it will not be presented here (2). However, arginine is also a vital nutrient for pathogens, supporting crucial processes such as protein synthesis and other cellular functions necessary for bacterial survival and growth (5, 6).

Therefore, it should be unsurprising that burgeoning evidence supports that arginine does more than serve as a metabolic substrate; it functions as a signaling molecule that regulates a range of bacterial processes (7–10). The ability of pathogens to sense and respond to arginine availability allows them to fine-tune bacterial fitness factors, ensuring that they adapt within the host environment. Bacterial fitness factors regulated in response to arginine include toxins, biofilm formation, secretion systems, and swarming motility (11–15). Arginine sensing and regulation of bacterial fitness factors have been reported in both Gram-positive and Gram-negative pathogens in a variety of host environments where it supports bacterial adaptation and persistence in the host (15–19). Arginine has also been reported to support bacterial immune evasion, which has been reviewed (1, 20, 21).

This review examines how arginine availability varies across different host niches and affects microbial behavior. We will specifically explore how bacterial species sense and respond to arginine. Ultimately, we will underscore the critical role of arginine as an exogenous cue regulating key microbial processes and highlight the intricate relationship between nutrient availability and bacterial fitness, which shapes the dynamics of host-pathogen interactions.

## ARGININE AVAILABILITY ACROSS HOST NICHES

Arginine concentrations vary significantly across different host tissues, influencing both cellular functions and pathogen behavior. The nutritional requirements for arginine in humans are met by dietary intake (4–6 grams per day), endogenous synthesis from citrulline (10%–15% of total arginine production), and protein turnover (80% of total circulating arginine) (22, 23). This section first outlines the absorption and synthesis of arginine and then provides an overview of the role of arginine in normal organ and tissue functions. Finally, we present how disruptions in arginine homeostasis are impacted during disease states. Understanding the tissue-specific variations in arginine levels provides context for understanding where arginine-induced bacterial phenotypes may occur within the host.

### Absorption and synthesis of arginine

**Fig 1:**
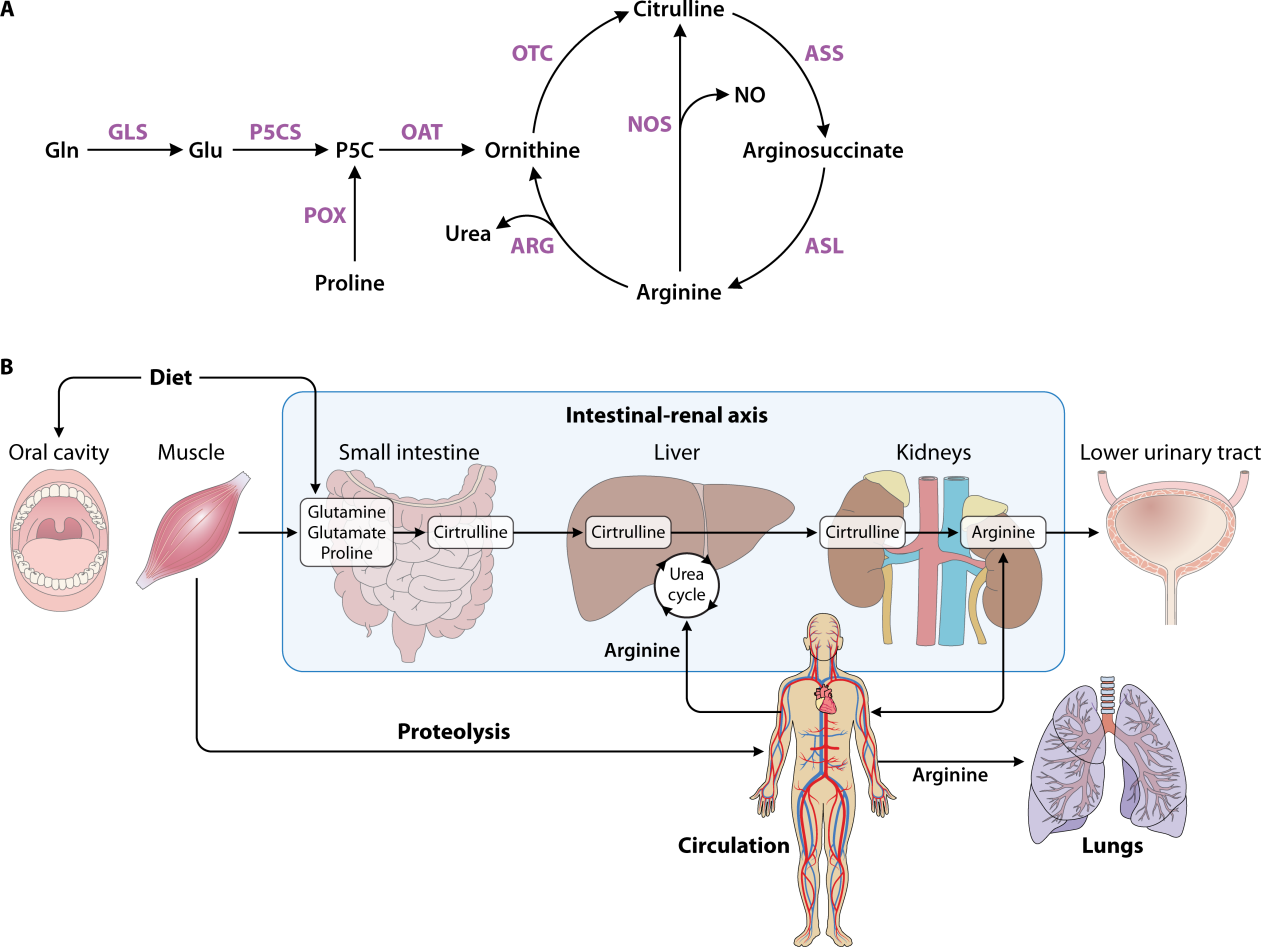
Overview of human arginine metabolism and availability in the human body. (**A**) Schematic representation of the human arginine synthesis pathway. Glutamine (Gln) is converted to glutamate (Glu), which is further processed to pyrroline-5-carboxylate (P5C). P5C is transformed into ornithine, which then is synthesized into citrulline. Citrulline goes into circulation and enters the kidneys where it is transformed to arginine. Key intermediates are shown in the context of the urea cycle. (**B**) Diagram depicting arginine availability in the human body, emphasizing its key sources and distribution. The intestinal-renal axis facilitates citrulline production in the intestines and conversion to arginine in the kidneys. Protein turnover contributes to endogenous arginine supply, while plasma circulation distributes arginine systemically. GLS = glutaminase; P5CS = pyrroline-5-carboxylate synthase; POX = proline oxidase; OAT = ornithine aminotransferase; OTC = ornithine transcarbamylase; ASS = argininosuccinate synthase; ASL = argininosuccinate lyase; ARG = arginase; NOS = nitric oxide synthase.

The small intestine is important for *de novo* arginine synthesis and absorption (22–24). In adult humans, dietary arginine is absorbed in the jejunum and the ileum of the small intestine by the y^+^ transporter. As meals can range in protein concentration, the dietary arginine levels found in the jejunum can range from ~230 µM (before a meal) to ~2,060 µM (3 hours after a protein-rich meal), while in the ileum, it ranges from 240 µM (before a meal) to ~400 µM (3 hours after a protein-rich meal) (Table 1) (25). However, ~40% of dietary arginine is degraded to ornithine by arginase (ARG) activity in the intestinal mucosa (Fig. 1A) (21, 23, 26–28). Additionally, the small intestine produces citrulline, which is important for renal synthesis of arginine (22, 24). In the small intestine, enterocytes expressing carbamoyl phosphate synthetase I and ornithine transcarbamoylase convert glutamine, proline, and ornithine into citrulline for delivery to targeted sites (Fig. 1A) (24). The critical role of the small intestine for absorbing dietary arginine is illustrated by the significantly reduced arginine concentrations in the plasma and muscles of rats with resected small intestines (29).

**TABLE 1:** Arginine concentrations in human compartments

Tissue/Organ	Arginine concentration	Reference(s)
Jejunum (small intestine)	~230–2060 µM	(25)
Ileum (small intestine)	~240–400 µM	(25)
Kidneys: synthesis	~10 mmol/day	(30, 31)
Kidneys: reabsorption	~18 mmol/day	(30, 31)
Plasma	6.7–210 µM	(21, 30, 32–34)
Intracellular	100–800 µM	(35–39)
Liver (hepatocytes)	30–100 µM	(21, 40)
Muscle	>1000 µM	(41, 42)
Urinary tract	10–200 µM; 2–13 µmol/mmol of creatinine	(43–45)
Oral cavity	~50 µM	(46, 47)
Sputum (CF)	0.3–300 mM	(48–52)

In humans, approximately 85% of intestinal citrulline released into circulation is absorbed in the proximal tubules of the kidneys (Fig. 1B) (22, 23, 30). In the kidneys, citrulline is converted to arginine by argininosuccinate synthetase and argininosuccinate lyase (Fig. 1A) (23). As the primary organ responsible for maintaining circulating serum arginine levels, the kidneys synthesize 10%–12% of the total arginine within the body (23, 53). Notably, not all cell types, such as immune cells, express every enzyme required for *de novo* arginine synthesis and therefore rely on circulating arginine for their functions (21). In a healthy kidney, the renal arginine synthesis is ~10 mmol per day (Table 1) (30, 31). In addition to synthesizing arginine, the proximal tubules in the kidneys are crucial for reabsorbing arginine from filtered plasma (~18 mmol per day) and returning it to circulation (Table 1) (30, 31). During filtration, <1% of the filtered load is lost in the urine and excreted (Fig. 1B) (30).

Once synthesized in the kidneys, arginine is released into the circulation, where it is transported to other body sites (Fig. 1B) (28, 54). Plasma is proposed to be the primary vehicle for amino acid exchange among tissues (41). In healthy individuals, the concentration of free L-arginine in human plasma fluctuates between 6.7 and 210 µM (Table 1) (21, 30, 32–34). However, the intracellular concentration of L-arginine is higher than in plasma (100–800 µM) (Table 1) (35–39). Thus, plasma circulation distributes arginine to various organs and cells (Fig. 1B).

Finally, the liver also plays an essential role in synthesizing arginine (55). Once the small intestine releases citrulline, it can enter the liver through the portal vein (Fig. 1B). It was previously thought that citrulline passed through the liver into circulation without any significant uptake (56–58). Recent findings show that the liver can absorb both circulating arginine and gut-derived citrulline (59). In non-cirrhotic fasting patients undergoing liver surgery, the splanchnic region—including the liver—was found to significantly remove arginine from systemic circulation, while hepatic uptake of citrulline limited the amount reaching the kidney, suggesting gut-derived citrulline can be absorbed from portal venous blood in the liver (59). The discrepancy in studies may be due to the limited accessibility of the portal vein in humans, with most human data coming from patients with liver disease and transjugular intrahepatic portosystemic shunts (60, 61). Regardless, the liver exhibits high ARG activity, converting arginine to ornithine and urea via the urea cycle (Fig. 1A). The urea is then excreted by the kidneys (62). In this way, most arginine absorbed or synthesized by the liver is eliminated via the urea cycle, with little to no arginine entering the plasma from the liver (23, 62). Essentially, liver arginine cycling is functionally separate from plasma arginine homeostasis. Arginine from the plasma can be taken up by the liver, but only ~5% of urea produced in the liver is from plasma arginine (Fig. 1B) (21). In fact, the liver is considered an arginine-depleted organ with concentrations being 0.03–0.10 µM in hepatocytes (Table 1) (21, 40). However, further research is needed to fully understand the role of the liver in the intestinal-renal axis.

### Protein turnover

Another significant source of arginine in the body is from protein turnover in muscle tissues (54). As skeletal muscle represents 40%–45% of adult body weight and is a protein-rich tissue, it is a crucial component in amino acid homeostasis (23, 63, 64). Specifically, intracellular free arginine is typically greater than 1 mM in muscle tissue (Table 1) (32, 42). The muscle tissue serves as a major source of L-arginine through proteolysis and is also thought to supply arginine as a precursor for NO synthesis during infection (Fig. 1B) (65).

### Functions of arginine during homeostasis

As a precursor to NO, peptides, and proteins, arginine plays a central role in maintaining tissue homeostasis and modulating the immune response. The following sections establish the availability and specific roles of arginine in both tissues and systemically during homeostasis.

Arginine plays a critical role in lung physiology, especially in immune defense against airborne pathogens. Pulmonary endothelial, epithelial, and infiltrated immune cells rely on circulatory arginine for high levels of NO synthesis (Fig. 1B) (66, 67). NO contributes to numerous physiological processes in the lungs, including airway and vascular smooth muscle relaxation, ventilation-perfusion matching, and host defense mechanisms such as bacteriostasis (66). However, the amino acid-rich bronchioalveolar fluid can also provide a nutritional source for bacteria like *Klebsiella pneumoniae* and *Pseudomonas aeruginosa* (68, 69).

The skin, as the primary barrier against external pathogens, also relies heavily on arginine to support immune function and wound healing. Arginine plays a crucial role in maintaining skin integrity through the ARG and inducible NOS (iNOS) pathways (70). The ARG pathway produces ornithine and, subsequently, polyamines, which are needed for collagen synthesis and cell proliferation, respectively (Fig. 1A) (70). Within a wound environment, arginine metabolism is complex; both skin cells and wound fluid—partially derived from macrophage autolysis—contain ARG, which facilitates healing (55). In parallel, skin-resident immune cells produce NO to combat pathogens that breach the barrier through wounds, leading to reduced local arginine concentrations compared to plasma (71). Furthermore, a murine burn wound infection model revealed that significantly depleted arginine levels in skin tissue were attributable to increased ARG activity by myeloid-derived suppressor cells (72). Taken together, these studies demonstrate that arginine contributes to skin barrier function and maintenance.

In the urinary tract, arginine concentrations are typically lower than in other tissues, like muscle or plasma. Reported levels in urine range from 10 to 200 µM, although factors such as hydration and diet cause fluctuations (Table 1) (43, 44). Thus, urine arginine levels have also been reported to range from 2 to 13 µmol/mmol of creatinine (Table 1) (45). As discussed in Section 2A, some arginine is excreted in the urine during renal filtration and reabsorption (Fig. 1B). Despite the lower levels of arginine in the urine, its conversion to NO is vital for supporting the normal function of the urothelium, smooth muscles, nerves, and blood vessels (73, 74). For example, a chronic bladder injury model in rats demonstrated that supplementing oral L-arginine significantly enhanced bladder tissue relaxation and prevented histopathological damage, possibly due to L-arginine-derived NO and improved mucosal blood flow (75). In addition to supporting bladder function, NO is required for immune cells to defend against urinary pathogens, such as *Escherichia coli* (75).

The oral cavity is a dynamic host niche with a diverse microbial community, influenced by factors such as nutrient availability and pH (46, 76). Saliva plays a critical role in maintaining oral homeostasis, serving as a reservoir of nutrients, antimicrobial compounds, enzymes, and buffers. Among the nutrients present in saliva, arginine has a dual role in microbial metabolism and oral health (46). Arginine is naturally present in saliva as a free amino acid and in salivary peptides and proteins (Fig. 1B). Physiological concentrations of arginine in free form in human saliva have been reported to be about 50 µM, depending on individual factors such as diet, salivary flow, and health status (Table 1) (46, 47). Salivary arginine is a substrate for the arginine deiminase (ADI) pathway, utilized by commensal oral bacteria to convert arginine to ammonia, neutralizing acids in the oral environment (76). This process is essential for buffering the pH and protecting against conditions like dental caries from pathogens like *Streptococcus mutans*.

Systemically, plasma L-arginine serves as a precursor for NO production, which is essential for physiological processes including vascular and immune functions (77–80). In human adults, NO synthesis drives 1.2% to 1.5% of plasma arginine flux. NO and L-citrulline are synthesized from L-arginine by endothelial NOS, and L-citrulline can be recycled back to L-arginine by the endothelial cells (Fig. 1A). NO regulates vasculature by dilating vascular smooth muscles via the guanylate cyclase pathway (78, 80). In pro-inflammatory conditions, such as infections, increased vasodilation and vascular permeability allow immune cells to infiltrate the site of infection or damage (81). Furthermore, NO is crucial for the killing of pathogens by macrophages and T-cell proliferation (4, 82). Thus, plasma arginine is crucial for the immune response, particularly during infections and recovery.

### Disruptions in arginine homeostasis during disease states

The role of arginine in homeostatic tissue function is disrupted during disease. This section examines how arginine levels fluctuate within distinct tissues—including the lungs, plasma and muscle, kidneys, intestine, and liver—and how it influences tissue-specific responses during a disease state.

Pulmonary arginine concentrations can vary between healthy and disease states. In diseases such as cystic fibrosis (CF), sputum levels of arginine (~0.3–300 mM) are often reported to be decreased, likely due to increased ARG I activity (48–52). This reduction in airway arginine is associated with diminished NO production, which has been linked to impaired lung function and heightened susceptibility to infection (83). However, other studies have reported elevated sputum and plasma arginine levels in pediatric CF patients, particularly during acute exacerbations, compared to healthy controls (84–86). These discrepancies may reflect differences in patient populations and disease state, as the studies reporting lower arginine levels excluded individuals experiencing acute respiratory symptoms. Overall, the interplay between arginine availability, ARG activity, and NO production highlights the important role of arginine in maintaining pulmonary health and defending against infections, especially in chronic inflammatory conditions like CF.

Plasma and muscle tissue are key components that maintain systemic arginine homeostasis (41, 65). During inflammatory states such as sepsis, arginine homeostasis in this compartment is disrupted, as evidenced by reduced plasma arginine concentrations (4, 87–89). For example, in a rat model of sepsis, both arginine and ornithine plasma concentrations decreased rapidly after lipopolysaccharide (LPS) stimulation in the early phase of sepsis (60 min) but rebounded as septicemia progressed (3–6 hours) (36). This suggests a dynamic regulation of plasma arginine throughout the course of sepsis. Notably, despite high intracellular L-arginine levels, the J774 macrophage cell line increased arginine import rates after LPS stimulation, highlighting the requirement of extracellular arginine (90). Similarly, endothelial cells produce NO from extracellular—not intracellular—L-arginine, reinforcing the critical role of circulating arginine (39). The mechanisms behind plasma arginine depletion during sepsis are likely a combination of decreased arginine uptake, impaired *de novo* synthesis from citrulline, and increased catabolism of arginine by ARG and NOS (88). Importantly, serum arginine is also depleted in other disease states—including cancer, liver injury or disease, or other severe traumas—likely due to increased ARG I activity and associated with impaired T-cell proliferation and cytokine production (82, 91–93). Concurrently, muscle is affected by increased protein breakdown during sepsis, contributing to muscle wasting (94, 95). In a porcine model, L-arginine supplementation decreased sepsis-induced muscle protein breakdown (4). Together, systemic arginine depletion during sepsis likely stems from both increased catabolism and impaired synthesis, with profound effects on immune function and muscle integrity.

The kidneys play a central role in arginine and citrulline metabolism, and their functions are significantly altered during both chronic kidney disease (CKD) and sepsis (31, 53). In cases of CKD, renal uptake of citrulline and release of arginine and other amino acids are reduced by 60%–70% (31, 53). The decreased release of arginine into serum likely affects NO levels, as NO production is reportedly lower in CKD (53, 96). Arginine and citrulline metabolism in the kidneys is also affected during sepsis. In a short-term mouse model of endotoxemia (modeling sepsis), LPS treatment increased net renal citrulline uptake and arginine release (56). In contrast, a rat model of endotoxemia reported no change in citrulline uptake, but renal arginine production was increased (58). These differing results on citrulline uptake may reflect differences in the timing of the infections (6 hours vs 16 hours, respectively). However, both studies reported increased renal arginine release, which could influence NO production or metabolites such as blood urea nitrogen. Together, kidney function influences arginine levels, with CKD reducing and sepsis generally increasing renal arginine production/release, thereby likely impacting systemic NO availability.

Intestinal levels of arginine have been shown to affect infection and inflammation in the gut. Studies have found oral arginine decreases mucosal injury caused by intraperitoneal LPS endotoxemia in a rat model and enhances bacterial clearance in an intestinal injury rat model (97, 98). In early-weaned pigs, higher doses of arginine (1.2%) exacerbated intestinal damage, reducing villus height and increasing diarrhea, compared to lower doses (0.7%) (99). These differences between studies could be due to differences in dosage, disease context, and species. These findings underscore the role of arginine in the intestines during disease, specifically that arginine can be both beneficial for repair and defense, yet harmful at high concentrations.

The liver is another key site where arginine metabolism is disrupted during disease. The liver contains the highest levels of ARG I in the body, reflecting its central role in the urea cycle (100). Therefore, liver damage or liver disease can cause hepatocytes to release ARG I into the circulating plasma (100, 101). The increased ARG I in the plasma results in a severe arginine deficiency in liver-related diseases; however, increased plasma levels of ARG do not always result in decreased arginine in other non-liver diseases, such as asthma (100–102). Overall, during injury or disease states, damaged hepatocytes release ARG, causing plasma arginine deficiency.

### Summary

Arginine plays a central role in human physiology and response to injury or infections. The small intestine contributes significantly to arginine homeostasis by absorbing dietary arginine and producing citrulline, a precursor for arginine synthesis in the intestinal-renal axis (Fig. 1B). The kidneys convert gut-derived citrulline into arginine, which helps maintain circulating levels essential for NO production, immune cell activity, and tissue healing. Meanwhile, the liver maintains its own supply of arginine, distinct from circulating levels. Beyond these systems, most circulating plasma arginine is derived from muscular protein breakdown, which supports tissue maintenance and immune defense at other organ sites. For example, arginine supports pulmonary immune defense, wound healing in the skin, and NO production during urinary tract infections and protects against dental caries in the oral cavity. The integrated distribution and metabolism of arginine throughout these tissues are essential for maintaining homeostasis and supporting recovery from injury or infection.

## BACTERIAL ARGININE SENSING AND RESPONSE

The ability of bacteria to sense and respond to arginine is crucial for their adaptation to varying environmental conditions, for example, in different environmental and host niches. Bacteria utilize biosensors such as ArgR and its paralogs, which serve as transcriptional regulators that bind arginine and modulate gene expression. This section highlights strategies bacteria use to sense and respond to arginine and the impact of arginine sensing on metabolism.

### Arginine sensing and response

Bacterial sensing of arginine primarily occurs through two known mechanisms: (i) transcriptional regulators, such as ArgR-family regulators, and (ii) cyclic diguanylate monophosphate (c-di-GMP) metabolizing enzymes containing arginine-binding domains such as Venus flytrap (VFT) or periplasmic substrate-binding protein (PBPb) domains (8–11, 42, 103–118). These two mechanisms allow bacteria to detect environmental arginine and regulate gene expression directly and indirectly.

ArgR-family regulators have been described in both Gram-negative species (e.g., *Salmonella typhimurium*, *E. coli*, *Legionella pneumophila*, *K. pneumoniae*, *P. aeruginosa*, and *Pseudomonas putida*) and Gram-positive species (e.g., *Bacillus subtilis*, *Enterococcus faecalis*, *Staphylococcus aureus, Streptococcus pneumoniae*, *Streptococcus suis*, and *S. mutans*) (42, 104–106, 108, 112, 113, 117–119). ArgR is primarily described as a direct sensor of L-arginine availability and transcriptional repressor of arginine biosynthetic genes (103). Upon binding L-arginine, the transcriptional regulator ArgR assembles into a hexameric complex that binds conserved DNA sequences in promoter regions known as ARG boxes (6, 103). Although ArgR-arginine complexes typically act as repressors, they can also activate gene transcription in some species (104–108, 119, 120). This dual regulatory role may be context-dependent, species-specific, or environmentally influenced.

To further complicate arginine-dependent regulation, bacteria can encode one to three ArgR paralogs (e.g., ArgR1, ArgR2, and arginine hydroxamate-resistant mutant C [AhrC]) (42). For example, AhrC is an ArgR homolog that also forms hexamers upon L-arginine binding, acting as a transcriptional repressor (103). These ArgR paralogs have been reported to regulate arginine metabolism pathways, including transport, biosynthesis, and catabolism (42, 113–117, 121). Thus, they likely support bacterial adaptations to changes in nutrient availability or host-associated cues.

**Fig 2:**
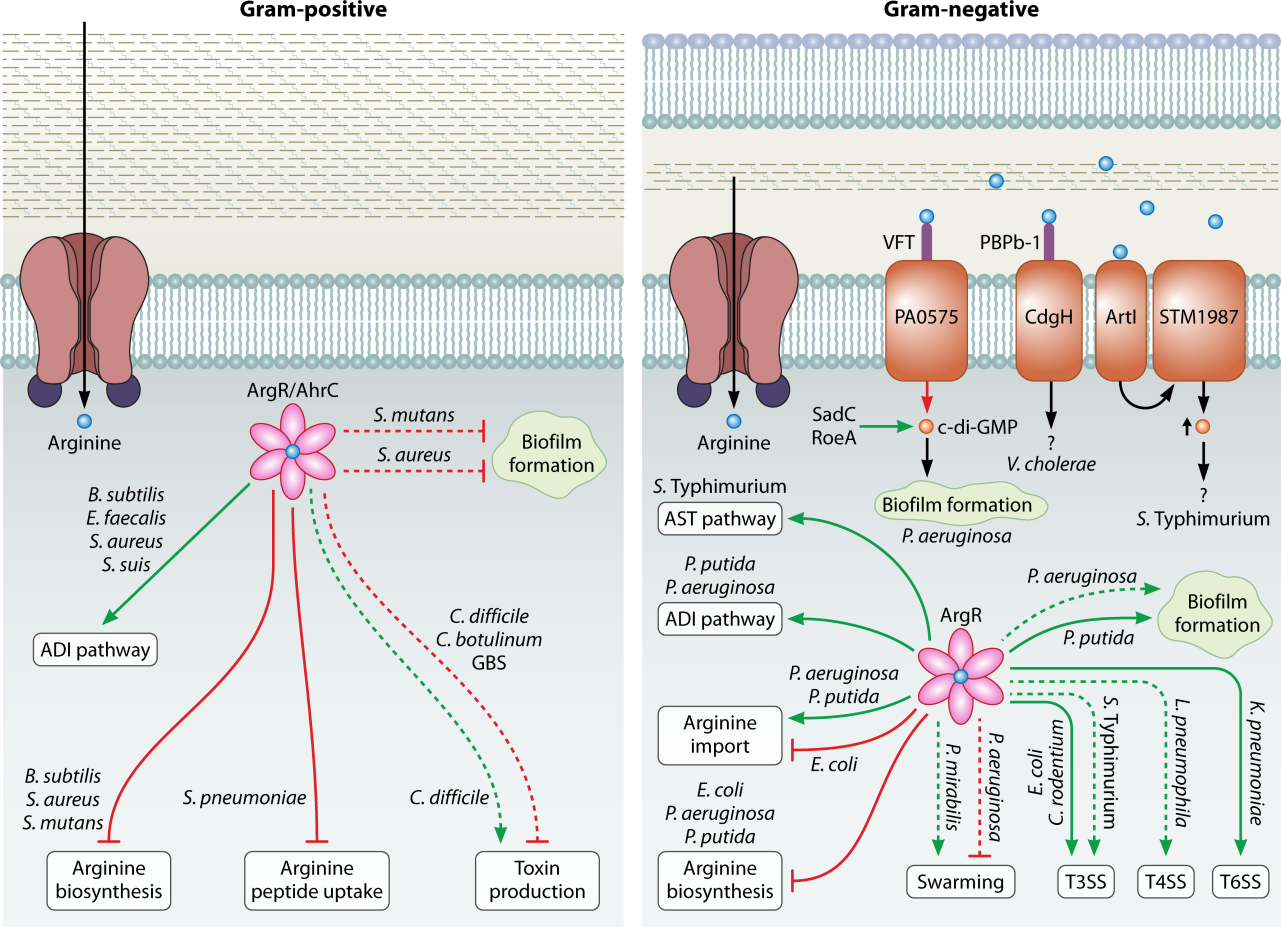
Arginine sensing mechanisms and regulation of bacterial fitness factors. Summary of arginine sensing mechanisms and ArgR regulation in gram-positive (left) and gram-negative (right) species. ArgR/AhrC functions as both a repressor and an activator depending on the bacterial species and context. Key bacterial fitness factors regulated by arginine across various bacterial species are illustrated, including biofilm formation, the T3SS, swarming motility, toxin production, Type IVA and Type IVB secretion systems (T4SS), and the T6SS. Additionally, ArgR/AhrC regulates a plethora of genes in multiple pathways including arginine biosynthesis, ADI pathway, arginine/peptide uptake/import, and the AST pathway. Regulation by the ArgR/AhrC complex is indicated by dotted lines for predicted regulation and solid lines for experimentally validated regulation. Green arrows identify positive regulation, and red arrows identify negative regulation. The orange boxes represent arginine sensing domains connected to c-di-GMP enzymes (VFT = Venus flytrap; PBPb = periplasmic substrate-binding).

The second major sensing mechanism involves c-di-GMP, a second messenger that regulates motility, biofilm formation, and virulence (8–10). Diguanylate cyclase (DGCs) and phosphodiesterase (PDEs) enzymes synthesize and degrade c-di-GMP, respectively. Some arginine sensing domains are connected to DGC or PDE domains, thus enabling bacteria to adjust intracellular c-di-GMP levels in response to arginine and indirectly regulate key behaviors. In *P. aeruginosa*, for example, the N-terminal VFT domain in the periplasmic region of the transmembrane protein PA0575 binds L-arginine (9). Strains lacking PA0575 have increased c-di-GMP levels when grown on L-arginine as the sole carbon source and ~12% higher adhesion (9). This supports the model that PA0575 decreases c-di-GMP in an L-arginine-dependent manner, likely as a PDE regulating *P. aeruginosa* adhesion (Fig. 2) (9). The inner membrane DGC enzyme, CdgH, in *Vibrio cholerae* binds L-arginine through two N-terminal tandem PBPb domains (Fig. 2) (8). Arginine-binding induces conformational changes in the CdgH dimer, but whether this binding alters CdgH enzyme activity and biofilm regulation needs further exploration (8). In *S*. Typhimurium, an enteric bacterium responsible for gastroenteritis, arginine sensing is coordinated by the inner membrane proteins ArtI and STM1987. ArtI is a PBP that binds and transports L-arginine into the cytoplasm, while STM1987, a DGC with a periplasmic Cache1 domain, responds to arginine to modulate c-di-GMP levels (Fig. 2) (10). Together, these studies demonstrate that periplasmic arginine is sensed through arginine-binding domains that modulate c-di-GMP-metabolizing enzymes, which can modulate intracellular c-di-GMP levels and alter bacterial behaviors, such as biofilm formation.

### Impact of arginine sensing on metabolism

The sensing of arginine by ArgR-family regulators has been reported to induce metabolic changes across multiple bacterial species. In this section, we examine how arginine sensing influences arginine biosynthesis and transport. We also examine how arginine sensing regulates arginine catabolic pathways such as the ADI pathway and the arginine succinyltransferase (AST) system, which break down arginine for energy or nitrogen. For each regulatory outcome, we describe the role of ArgR-family regulators and highlight species-specific differences in their function.

ArgR-family regulators have been repeatedly reported to repress arginine biosynthetic genes and activate import in several species. In *P. aeruginosa*, ArgR represses three key enzymes in the arginine biosynthetic pathway encoded by *argF*, *carAB*, and *argG* in the presence of L-arginine (Fig. 2) (111). A similar pattern is observed in *P. putida* (now *Pseudomonas alloputida*)*,* where ArgR negatively regulates arginine biosynthesis, primarily by controlling the expression of *argG* (112, 121). Similarly, in *S. mutans*, AhrC negatively regulates arginine biosynthesis (Fig. 2) (118). Taken together, these findings are consistent with the classical role of ArgR as a repressor of arginine biosynthetic operons in the presence of arginine (103).

In addition to arginine biosynthesis repression, ArgR also positively regulates the *aotJQMOP-argR* operon, which controls arginine and ornithine import, and ArgR itself in *P. aeruginosa* (110). Similarly, in *P. putida*, ArgR positively regulates arginine import via *argT* (112, 121). In contrast, in *E. coli,* ArgR represses arginine import genes and biosynthesis genes (103). While in *S. pneumoniae*, ArgR1 and AhrC control the expression of genes involved in arginine and peptide uptake. Together, these findings exemplify that ArgR-family regulators can also coordinate arginine, ornithine, and peptide acquisition in a species-specific manner (Fig. 2) (42).

Arginine catabolism occurs through the AST and ADI pathways. *S. typhimurium* has an *ast* operon which encodes the AST system that converts arginine to glutamate, CO2, and ammonia, as a nitrogen source (104, 105). Exogenous arginine induces the *ast* operon in nitrogen-limited conditions, which is abolished in an *argR* mutant, suggesting that ArgR regulates the *ast* operon in response to arginine (Fig. 2) (104). Similarly, in *P. putida,* genes in both the AST and ADI pathways are upregulated by ArgR (Fig. 2) (104, 105). In contrast, in *E. coli*, ArgR is not essential for *ast* operon expression, highlighting species-specific differences in ArgR regulons (105).

The bacterial ADI pathway catabolizes arginine into ornithine, carbamoyl-phosphate, ATP, CO_2_, and ammonia. The ADI system is usually upregulated under anaerobic conditions and downregulated during the stringent response, a global stress response that prioritizes survival over growth (108, 122). In *P. aeruginosa*, ArgR positively regulates expression of the genes involved in the ADI pathway to generate ATP from arginine during anaerobic conditions (Fig. 2) (108, 123). ArgR binds to a conserved ArgR-binding sequence located upstream of the transcriptional anaerobic regulator (ANR) binding site in the *arcD* promoter (108). Expression of the ADI pathway is not induced by arginine in the absence of ANR, suggesting that ArgR requires ANR to initiate transcription (108). Similarly in *S. suis*, a major porcine pathogen, ArgR activates the ADI (*arc*) operon, which is essential for acid resistance and bacterial fitness (Fig. 2) (117). Arginine also induces the ADI operon (*arcDABC*) in *E. faecalis,* likely via ArgR1/ArgR2 binding to predicted ARG boxes in the *arcA* promoter (Fig. 2) (*114).* Given that 40% of dietary arginine is converted to ornithine in the gastrointestinal tract and *E. faecalis* is a common gut colonizer, it is possible that *E. faecalis* activates the ADI pathway to degrade intestinal arginine to ornithine and ammonia in the human gut. Finally, in *B. subtilis* and *S. aureus*, AhrC represses arginine biosynthesis and activates arginine catabolism, supporting the switch from synthesis to degradation in response to elevated arginine availability (Fig. 2) (113, 115). Altogether, these examples highlight how ArgR-family regulators integrate arginine sensing with the control of arginine metabolism in changing environmental or host conditions.

## REGULATION OF BACTERIAL FITNESS FACTORS BY ARGININE

Upon sensing changes in arginine concentrations, bacteria have been reported to regulate several cellular processes that enable their survival and pathogenicity in the host. These processes include biofilm formation, secretion systems, swarming motility, toxin production, polymicrobial interactions, and host-related adaptation, persistence, and immune evasion.

### Biofilm formation

Biofilm formation supports bacterial colonization and survival by enabling a transition from a motile, planktonic lifestyle to a structured, surface-attached community encased in an extracellular matrix (124). The biofilm state protects bacteria against environmental stressors, including immune responses and antimicrobial agents (124). Bacterial species like *P. aeruginosa*, *P. putida*, *S. aureus*, and *S. mutans* are well-known for their ability to form biofilms in diverse environments ranging from medical devices to host tissues.

Arginine has emerged as a key modulator of biofilm formation in *P. aeruginosa*. In the PA14 strain, arginine promotes biofilm formation and suppresses swarming motility (Fig. 2) (51). Additionally, physiological concentrations of arginine (50 and 100 mM) combined with tobramycin promote *P. aeruginosa* biofilm formation (50). The DGCs, SadC and RoeA, are required for arginine-dependent increases in biofilm formation and decreases in swarming motility, likely through modulation of c-di-GMP levels in the PA14 strain (51). Although the sensing domains of SadC and RoeA remain uncharacterized, these findings suggest that arginine availability, especially in combination with antibiotics, could increase *P. aeruginosa* biofilm formation. Conversely, in burn wounds—where skin arginine concentrations are reduced—*P. aeruginosa* swimming motility increases but can be reduced when exogenous arginine (>100 mM) is supplemented (72). Together, these studies suggest that arginine availability could fine-tune *P. aeruginosa* behavior in different infection settings.

Similarly, in *P. putida*, ArgR is essential for concentration-dependent increases in c-di-GMP levels in response to arginine, which enhance biofilm biomass and decrease motility (Fig. 2) (112, 125). Arginine import appears essential to this phenotype, as mutants lacking the arginine transport proteins, ArgT and ArtJ, partially lose the arginine-dependent increase in c-di-GMP levels, while arginine biosynthesis mutants (*argG* and *argH*) exhibit reduced c-di-GMP that can be partially restored by exogenous arginine (125). Furthermore, ArgR fine-tunes biofilm composition in *P. putida* by repressing LapF and Pea, which promote cell-cell adhesion and exopolysaccharide production, while activating the *bcs* operon to increase cellulose production, suggesting that different types of biofilms form depending on environmental cues (121). Thus, *P. putida* appears to coordinate biofilm formation and type in response to cellular arginine pools via both c-di-GMP- and ArgR-dependent regulatory loops.

In contrast, the role of arginine in *S. aureus* and *S. mutants* biofilm formation is more variable and context-dependent. In the *S. aureus* strain UAMS-1, the ADI cluster (*arcABDC*) is upregulated in biofilm conditions, but a ∆*arcD* (arginine catabolism) mutant strain achieved similar *in vivo* cell densities as the wild-type strain in a mouse model of indwelling device infections (Fig. 2) (126–128). This suggests that while arginine catabolism is associated with biofilm growth, its direct role in biofilm establishment or maintenance is unknown. In *S. mutans*, arginine promotes biofilm formation at low concentrations (5 and 10 µM) but reduces it at higher concentrations (100 µM) (Fig. 2) (129). This regulation is likely mediated by AhrC, a negative regulator of biofilm formation in response to arginine (118). Given that the physiological concentration of arginine in the oral cavity is approximately 50 µM, further research is needed to evaluate how saliva arginine concentrations directly impact *S. mutans* attachment and biofilm formation in this host niche.

Together, these findings highlight that arginine can act as a key environmental cue modulating biofilm formation across diverse bacterial species to regulate biofilm formation depending on the organism and arginine concentration. Additional studies reporting arginine-linked biofilm modulation in these aforementioned pathogens are listed here (130–132). Altogether, these examples underscore the importance of arginine availability and sensing in shaping bacterial behavior and biofilm-associated persistence during host colonization and infection.

### Secretion systems

Bacterial secretion systems are used to transport proteins across cellular membranes to interact with host cells, compete with other microbes, or modify the extracellular environment (133). Arginine has been reported to regulate the Type III, Type IV, and Type VI secretion systems (T3SS, T4SS, and T6SS), which are particularly important for pathogenesis and interbacterial competition.

Arginine availability influences T3SS expression in several bacterial species, including enterohemorrhagic *E. coli* (EHEC), *Citrobacter rodentium*, and *S*. Typhimurium (Section 4E). EHEC virulence determinants include the locus of enterocyte effacement (LEE) pathogenicity island, which encodes a T3SS and the Shiga toxin (Stx2a) (11). One study using RNA sequencing demonstrated that exogenous arginine upregulated the expression of LEE-encoded genes via ArgR in both EHEC and *C. rodentium*, a murine pathogen (11). Furthermore, they showed that in the presence of arginine, ArgR directly activates the expression of genes encoding the T3SS (Fig. 2) (11). These findings highlight the critical role of exogenous arginine in regulating the virulence mechanisms of EHEC and *C. rodentium*, providing potential avenues for therapeutic intervention.

The regulation of the T4SS in *L. pneumophila* is closely tied to arginine sensing via an *argR* homolog (109). The *argR* homolog (*lpg0490*), a σ^S^-regulated gene, is crucial for optimal intracellular multiplication of *L. pneumophila* within the protozoan host *Acanthamoeba castellanii,* but not in THP-1 macrophage cell lines (109). ArgR senses arginine levels in the *Legionella*-containing vacuole (LCV) and represses gene transcription but de-represses transcription during intracellular growth (106). This suggests that the LCV contains sufficient L-arginine to support growth since *L. pneumophila* is an arginine auxotroph. Rather than regulating arginine biosynthesis, ArgR represses components of the Type IVA and Icm/Dot Type IVB secretion systems during post-exponential growth (Fig. 2). The Icm/Dot system transports effector proteins into host cells, helping the bacteria evade phagolysosome fusion (106). Notably, ArgR positively regulates 17 out of 140 genes encoding Icm/Dot-translocated proteins, suggesting it plays a role in regulating other proteins crucial for infection (106). Taken together, this suggests the *argR* homolog could be important for the timing and assembly of the Type IVB secretion system in *L. pneumophila*.

The T6SS in *K. pneumoniae* is required for gut colonization and regulated by ArgR in response to arginine availability (Fig. 2). *K. pneumoniae* strain KPPR1S encodes two T6SS loci, and arginine significantly increases the expression of both loci (120). This upregulation is mediated by ArgR, which binds to an ARG box located within the promoter region of the T6SS-1 promoter (120). Functionally, the T6SS delivers toxic effectors to neighboring bacteria, allowing *K. pneumoniae* to outcompete commensal microbes in the gut. Thus, the presence of arginine in the gastrointestinal (GI) tract serves as a key signal for *K. pneumoniae* to activate its T6SS, enhancing its ability to establish gut colonization and overcome host-mediated colonization resistance.

### Swarming

Swarming motility, the coordinated multicellular movement across surfaces, is a key behavior enabling bacterial colonization and infection (134). This specialized motility is tightly regulated by environmental cues, including the availability of amino acids such as arginine (134). In *Proteus mirabilis*, a leading cause of catheter-associated urinary tract infections, the addition of 0.1 mM arginine to agar plates prepared from pooled human urine significantly enhanced swarming motility without affecting swimming motility or bacterial growth rate (Fig. 2) (15). In swarming, the flagellar rotation is dependent on the proton motive force (µH^+^) to increase energy (16). Arginine catabolism generates energy and increases the µH^+^ (16). Therefore, it is not surprising that arginine availability enhances *P. mirabilis* swarming motility by increasing the µH^+^. In contrast, *P. aeruginosa* demonstrates a different response to arginine. While *P. aeruginosa* is capable of swarming motility, studies have shown that when arginine is the sole nitrogen source, it does not swarm but instead favors biofilm formation (as described above in Section 4A) (Fig. 2) (51, 130). These findings highlight how arginine availability modulates swarming motility in a species-specific manner, likely influencing colonization and pathogenesis. It is possible that niche-specific demands have selected for differential effects of arginine on bacterial control of motility versus adhesion.

### Toxin production

Toxin production is a hallmark of bacterial pathogenesis. Thus far, arginine has been shown to regulate exotoxin production in *Clostridium botulinum*, *Clostridioides difficile*, and *Streptococcus agalactiae* (Group B *Streptococcus* [GBS]).

Arginine modulates botulinum neurotoxin production in *C. botulinum*. Early studies reported that *C. botulinum* strains that produce Type A or B neurotoxin require arginine for growth (135–138). While certain nutrients, such as glucose, tryptophan, and casein digests, increased *C. botulinum* toxin production, the titrated addition of arginine to defined medium reduced protease and toxin production (Fig. 2) (139). Many investigators who studied *C. botulinum* were reportedly frustrated by sporadic toxin titers (139). It is possible that the dual nature of arginine supporting *C. botulinum* growth yet suppressing toxin production, combined with media batch variations, wrought these challenges. Further research is needed to investigate how arginine concentrations in the human GI tract influence *C. botulinum* toxin production.

Arginine availability also shapes *C. difficile* toxin production. *C. difficile*, a gram-positive bacterium and a leading cause of gastrointestinal infections, relies on the production of two potent toxins, TcdA and TcdB, which drive intestinal epithelial cell death and inflammation (12). Multiple studies have shown that metabolic cues, including arginine, influence toxin production. One study utilizing Phenotype MicroArrays (Biolog) found that arginine and multiple arginine dipeptides were potent inducers of toxins in the *C. difficile* ATCC 9689 strain (Fig. 2) (13). Similarly, a second study showed that culturing *C. difficile* VPI 10463 strain in trypticase-yeast extract medium supplemented with both arginine and glucose led to the highest toxin titers observed (10^6^ cytotoxin titers at 72 hours) (14). However, a third study showed that three strains (KZ 1630, KZ 1647, and KZ 1748) had increased toxin production in the absence of arginine, although these strains exhibited impaired growth under that condition (140). The discrepancies between studies could be due to the different strains used and/or tested conditions.

The expression of GBS hemolysin is also influenced by arginine (122). GBS is a gram-positive bacterium that can cause neonatal sepsis, pneumonia, and meningitis. The ornithine-rhamnopolyene β-hemolysin/cytolysin (βHC) is a well-studied GBS virulence factor that is cytotoxic to a variety of human cells (122). One study demonstrated that incubating the GBS strain, A909, with arginine increased hemolysis and that the arginine structural analog, canavanine, suppressed hemolysis (122). The stringent response usually decreases the expression of genes in the ADI pathway (arginine catabolism) (122). Thus, the GBS stringent response leads to arginine-dependent increases in βHC production (Fig. 2) (122). This highlights how arginine availability can increase GBS virulence. All together, these findings underscore the complex and context-dependent role of arginine in modulating toxin production across diverse bacterial pathogens.

### Polymicrobial interactions

The dynamics of arginine-dependent signaling become more complex when one considers that microbes usually exist in mixed populations. Polymicrobial communities are notable in host sites, like the oral cavity, GI tract, and urinary tract. In such niches, interspecies interactions can influence infection outcomes. In this context, arginine levels have been reported to serve as a cue that changes bacterial behavior and virulence. Below, we provide three examples where arginine dynamics between *Candida albicans* and *Salmonella*, *C. difficile* and *E. faecalis*, and *P. mirabilis* and *E. faecalis* have been reported to alter bacterial pathogenesis.

Recently, arginine release in the gut microenvironment was found to exacerbate *Salmonella* pathogenesis. Specifically, arginine-driven interactions between the fungus *C. albicans*, a common gut colonizer, and *Salmonella* were found to enhance *Salmonella* gut colonization and systemic dissemination (141). The delivery of *Salmonella* SopB to *C. albicans* via the T3SS increased *C. albicans* arginine biosynthesis, resulting in millimolar concentrations of arginine in the extracellular environment. This extracellular arginine then induced *Salmonella* T3SS expression, facilitating epithelial cell invasion (Fig. 2) (141). Additionally, arginine supplementation in the drinking water increased the systemic spread of *Salmonella* in a mono-gut colonization model (141). Supporting these findings, *Salmonella* is also known to access host cytosolic arginine reservoirs by recruiting the host cationic amino acid transporter (mCAT1) to the *Salmonella*-containing vacuole (SCV) to support its growth (2). Together, these findings underscore the pivotal role of arginine as a key metabolite mediating trans-kingdom interactions between host, bacteria, and fungi.

While arginine has been shown to modulate *C. difficile* toxin expression (Section 4D), interactions with other gut microbes—such as *E. faecalis—can*—can alter local arginine availability and further influence *C. difficile* virulence. These findings were developed based on clinical data that *E. faecalis* is enriched in the stool of pediatric patients experiencing *C. difficile* infections (142). It was previously established that *E. faecalis* depletes arginine via the ADI pathway to produce ornithine (Fig. 2). The working model from this study is that *E. faecalis* increases *C. difficile* pathogenicity by decreasing arginine and increasing ornithine levels in the gastrointestinal tract. In both *in vivo* and *in vitro* models, *E. faecalis* conversion of arginine to ornithine enhanced *C. difficile* CD196 pathogenesis and toxin output (Fig. 2) (142). These clinical data align with the earlier findings that *C. difficile* toxin production increases under arginine-limited conditions despite impaired growth. Overall, the interaction between *C. difficile* and *E. faecalis* highlights the central role of arginine in enteric bacteria and, specifically, in *C. difficile* toxin production.

Interbacterial arginine dynamics have also been reported to alter bacterial pathogenesis in the urinary tract. *E. faecalis* and *P. mirabilis* are frequent co-colonizers in catheterized patient populations and have an enhanced capacity to form biofilms (143). Recent work has shown that arginine/ornithine exchange between *P. mirabilis* and *E. faecalis* drives enhanced biofilm formation on catheters (144). Co-culturing *P. mirabilis* with an *E. faecalis arcD* (arginine/ornithine antiporter) mutant reduced biofilm biomass, whereas co-culturing a *P. mirabilis argI* (arginine biosynthesis) mutant with wild-type *E. faecalis* still enhanced biofilm formation (144). Additionally, co-culturing wild-type or *argI P. mirabilis* with *E. faecalis arcD* supplemented with 10 mM L-arginine restored biofilm formation. (144) Taken together, these data support that *E. faecalis* secretes L-ornithine via ArcD and fuels arginine biosynthesis and metabolism in *P. mirabilis*, which increases biofilm mass.

Overall, arginine plays a crucial role in mediating interspecies interactions within polymicrobial communities, enhancing bacterial persistence, biofilm formation, and virulence across various host niches. *E. faecalis* has been repeatedly reported to drive arginine-ornithine polymicrobial dynamics with *C. difficile* in the gut, *P. mirabilis* on urinary catheters, and with *E. coli* in wound infections (142, 144, 145). Whether facilitating cross-kingdom interactions in the gut or driving biofilm enhancement in the urinary tract, polymicrobial arginine exchange significantly influences infection outcomes.

### Adaptation, persistence, and evasion in the host

Arginine sensing and metabolism are central to the ability of diverse bacterial pathogens to adapt to hostile host environments, persist within host niches, and evade immune defenses. Across species—including *Francisella tularensis*, *Streptococcus pyogenes*, *S*. Typhimurium, *E. coli*, and *S. aureus*—arginine supports key adaptive functions such as intracellular replication, acid resistance, oxidative stress tolerance, immune evasion, or niche-specific persistence.

Arginine acquisition is essential for intracellular survival and replication in several pathogens. In *F. tularensis*, obtaining arginine from the host is critical for its intracellular survival in macrophages (17). Both *F. tularensis* subsp. novicida and *F. tularensis* subsp. holarctica live vaccine strain (LVS) require the arginine transporter ArgP for phagosomal escape and intracellular multiplication (17). Similarly, *S. pyogenes* (group A *Streptococcus*) invades and survives within a variety of host cells and also relies on arginine for intracellular survival (18, 146–154). The production of streptococcal acid glycoprotein (SAGP), which has arginine deiminase activity, facilitates resistance to intracellular acidification (18). *S. pyogenes* Manfredo strain lacking SAGP has impaired ability to invade and survive intracellularly, but supplementation with arginine (≥1 mM) restores survival (18). Thus, for these two species, intracellular survival and persistence depend on arginine acquisition.

Arginine availability and metabolism play a critical role in the acid stress responses of enteric pathogens (155–157). The acid resistance system 3 (AR3) neutralizes low pH with an arginine decarboxylase (AdiA) and arginine-agmatine antiporter (155). Accordingly, exogenous arginine enhances *Salmonella* acid tolerance in multiple strains under anaerobic conditions, while the absence of arginine results in acid sensitivity (155). Similarly, in *E. coli*, AR3 is arginine-dependent and relies on acid-inducible AdiA for survival in gastric acidity (157, 158). Loss of *adiA* abolishes resistance at pH 2.5, and mutations in *adiC*, encoding the arginine:agmatine antiporter, likewise disrupt AR3 function (157). In contrast, density-dependent acid survival in *E. coli* strains K-12 and O157:H7 correlates with limited arginine and glutamate availability at high cell densities (159). Beyond acid resistance, arginine metabolism also supports intracellular survival within epithelial cells and macrophages, where *Salmonella* resides in the SCV. A mutant lacking the *argCBH* operon, essential for L-arginine biosynthesis, was hypersensitive to hydrogen peroxide, less virulent in wild-type mice, and regained virulence in Cybb^⁻/⁻^ mice lacking NADPH oxidase (160). This sensitivity was rescued by pre-growth in high concentrations of arginine (460 mM) and linked to a collapse in intracellular pH rather than changes in respiration, highlighting the role of arginine in pH homeostasis and protection against host-derived reactive oxygen species (160). Together, these findings highlight a frequent reliance on arginine metabolism and transport mechanisms for acid resistance and stress adaptation among enteric pathogens.

Uropathogenic *E. coli* (UPEC) exemplifies host-adaptive metabolic flexibility during urinary tract colonization. Genomic analyses of the UTI89 strain identified genes under positive selection, including *argI*, which encodes ornithine transcarbamoylase, a key enzyme in arginine biosynthesis (161). In a murine model of chronic cystitis, mutants lacking *argI*, *argA*, or *argG*—all involved in arginine biosynthesis—exhibited arginine auxotrophy and reduced fitness during persistent infection (162). However, only *argI* showed evidence of lineage-specific positive selection within the B2 phylogroup of *E. coli*. Allelic replacement experiments demonstrated that the UTI89 *argI* allele conferred a competitive advantage over the non-B2 *argI* allele from strain MC4100, suggesting a specialized role in pathogenesis. In addition to supporting arginine biosynthesis, *argI* may also enhance polyamine production, contributing to UPEC persistence in the urinary tract (162). These findings underscore the evolutionary refinement of arginine metabolism as a strategy for niche adaptation and chronic infection in UPEC.

In *S. aureus* USA300, the constitutively expressed arginine catabolic mobile element (ACME)-encoded arginine-deiminase (Arc) system is necessary and sufficient for growth in acidic, skin-like conditions (pH 5.0) (163). Unlike the chromosomal Arc system, ACME-Arc functions independently of glucose or oxygen, enabling acid tolerance in aerated environments like the skin (163). In a skin and soft tissue infection model, ACME-Arc activity was associated with elevated host polyamine production and decreased iNOS levels in abscesses, suggesting that ACME-Arc diverts host arginine away from NO synthesis and toward polyamine pathways (163). Although polyamines promote persistence, their accumulation is toxic to *S. aureus*, requiring SpeG for detoxification. A Δ*speG* mutant is significantly less viable than wild-type by day 7 in abscesses, but ACME-Arc inactivation in the Δ*speG* background restored persistence, highlighting the interplay between arginine catabolism and polyamine resistance (163). These findings underscore a dual role for arginine catabolism in environmental adaptation and immune interference.

Together, these findings illustrate how arginine acquisition and metabolism contribute to pathogen survival across varied host environments—whether by supporting acid resistance, evading phagocyte-mediated killing, or facilitating chronic tissue colonization.

## CONCLUDING REMARKS

While our understanding of systemic arginine metabolism is well-established, the local arginine availability within specific tissues or organs, such as the liver or urinary tract, is not as well understood. Key questions include how inflammation alters arginine distribution between and within host organs, how these arginine variations impact host immunity and microbial adaptation, and whether arginine depletion is a passive consequence of infection or an active host defense mechanism. Advancing tools for *in situ* measurement of arginine and its related metabolites, such as high-resolution spatial metabolomics, could help define how arginine availability shifts during infection and, subsequently, how arginine dynamics between host and pathogen shape infection outcomes (142).

Yet to fully capitalize on these insights, it is equally important to understand the microbial aspect—namely, how bacteria sense and adapt to fluctuating arginine levels within the host environment. Traditionally, ArgR has been characterized as a negative regulator, particularly of arginine biosynthesis genes. However, emerging studies suggest that in certain pathogens, ArgR also functions as a positive regulator. The mechanisms underlying this dual regulatory function remain unclear and could vary between species or environmental contexts. Further investigation is needed to determine whether this functional versatility is more widespread and how it is mechanistically achieved.

Beyond transcriptional control, a major gap remains in understanding how arginine influences bacterial fitness—such as biofilm formation, toxin production, immune evasion, and metabolic adaptation—in different host environments. Many pathogens rely on arginine-responsive regulators to fine-tune their virulence in response to nutrient cues, yet we know little about where within the host these systems are activated or suppressed. Understanding host arginine dynamics and bacterial arginine-dependent regulation during colonization and infection could uncover how tissue-dependent differences in arginine shape the course of bacterial pathogenesis.

Ultimately, arginine represents more than just a nutrient—it serves as a key regulatory signal at the host-pathogen interface. Further defining how arginine abundance changes between colonization and infection states and how this shapes bacterial growth and virulence programs is critical. By understanding how host arginine distribution shapes bacterial pathogenesis, we can uncover new strategies to prevent infection. Mapping these arginine-responsive pathways *in vivo* will be critical for identifying vulnerable stages of infection that could be amenable to therapeutic intervention.
